# Statistics as a Social Activity: Attitudes toward Amalgamating Evidence

**DOI:** 10.3390/e26080652

**Published:** 2024-07-30

**Authors:** Andrew Gelman, Keith O’Rourke

**Affiliations:** 1 Department of Statistics Columbia University, New York, NY 10027, USA; 2 Department of Political Science, Columbia University, New York, NY 10027, USA; 3 O’Rourke Consulting, Ottawa, ON K1P 6K7, Canada

**Keywords:** Bayesian inference, foundations of statistics

## Abstract

Amalgamation of evidence in statistics is conducted in several ways. Within a study, multiple observations are combined by averaging, or as factors in a likelihood or prediction algorithm. In multilevel modeling or Bayesian analysis, population or prior information is combined with data using the weighted averaging derived from probability modeling. In a scientific research project, inferences from data analysis are interpreted in light of mechanistic models and substantive theories. Within a scholarly or applied research community, data and conclusions from separate laboratories are amalgamated through a series of steps, including peer review, meta-analysis, review articles, and replication studies. These issues have been discussed for many years in the philosophy of science and statistics, gaining attention in recent decades first with the renewed popularity of Bayesian inference and then with concerns about the replication crisis in science. In this article, we review the amalgamation of statistical evidence from different perspectives, connecting the foundations of statistics to the social processes of validation, criticism, and consensus building.

## 1. Aggregating Information in a Social Context

Weighing and amalgamating evidence is a central problem in the process of science, giving rise to much debate on what methods are appropriate as well as exactly where, when, and for what purposes they should be used. Within statistics, a central area of controversy is how to incorporate prior information in the context of objective and reproducible science. On the other hand, the weighing and amalgamating of evidence within a single isolated study (the multiple observations) in many default approaches in statistics is (surprisingly) often just automatic and implicit.

Vigorous debate on basic approaches in statistics likely comes as no surprise to statisticians and, increasingly, almost everyone else. Although there is much agreement on the mathematical definitions of terms and procedures in statistics (what they are) and on discerning if particular instances meet these standards (is it this?), the appropriate roles for these terms and procedures in facilitating scientific inquiry—their very purposes and what to make of them—seem beyond agreement for the foreseeable future. The tools are largely agreed upon, but their appropriate use, where, and for what purposes, are not at all. For instance, there is a fair amount of agreement on what probabilities are, but not on what they can be used for. Many frequentists ban any use of probability in representing (uncertain) knowledge of unknown parameters. On the other hand, while nearly all Bayesians utilize probabilities to represent knowledge (or lack of it), some would oppose any form of testing or empirical assessment of these. In the case of a single study, some statisticians would be concerned about the properties of procedures that can be discerned if the procedure would be repeatedly applied infinitely often under similar kinds of studies or even exactly the same study conditions. Others argue this is not even sensible.

Gillies [1,2] discussed a “pluralist” or “intersubjective” interpretation of probability, which is related to the concept of “institutional decision analysis”, for which an important aspect of inference is that decisions be justifiable, which motivates clear linkages between measurement, modeling assumptions, inferences, and decision recommendations. Once we situate science and data analysis in a social context, these challenges become clear. In the present paper, we do not set up any agent-based models; rather, our goal is to provide a broader perspective on the problem of aggregation of evidence in statistics.

Going beyond a single isolated study, the system of scientific publication, criticism, and meta-analysis provides more general avenues for the amalgamation of evidence rather than just within a study, and here, individual statistical analyses can be understood as (first) steps in this larger process. Perhaps unsurprisingly in this larger process, disagreements abound as opinions vary on what contextual (extra-study) information can be incorporated, where, and how. Should previous studies be amalgamated in a combined analysis, used to build a judgment-informed prior, or merely used qualitatively to refine the analysis, helping the study to stand on its own as much as possible? In this article, we present a general perspective on statistics as primarily about conjecturing, assessing, and adopting idealized representations of reality, predominantly using probability-generating models for both parameters and data. This approach involves using an explicit prior probability distribution to represent available but rough scientific conjectures about what values the unknown parameters might have been set to, and a data-generating probability distribution to represent how the recorded data likely came about if the unknown parameters’ values were set to specific possible values. This contrasts with another perspective on statistics, which views it primarily as a way to discern procedures with good properties that are uniform across a wide range of possible underlying realities and restricts their use, especially in science, to just those procedures. Our perspective is more conducive to information aggregation, as reality likely contains many commonalities that can be discerned and profitably utilized. We believe this approach can unify seemingly distinct statistical philosophies and also offer guidance in resolving the current replication crisis in science. When claims fail to replicate, the methods used likely did not reflect reality well, if at all.

## 2. Statistics as Amalgamation of Evidence

One of the frustrating—and fascinating—aspects of statistics, compared to many other modern sciences, is its profusion of seemingly incompatible philosophies. The Neyman–Pearson approach focuses on defining procedures for discriminating between hypotheses, targeting uniform type I errors for all null hypotheses and uniformly minimum type II errors for all alternatives. The Fisherian *p*-value, in contrast, evaluates the strength of evidence against a single null hypothesis without explicit reference to any alternative, targeting a uniform (0,1) distribution of *p*-values for all nulls. Another Fisherian approach, maximum likelihood, provides estimates within a parametric model (see [3,4,5]); meanwhile, Neyman–Pearson testing can be interpreted in Bayesian terms [6,7]. Bayesian inference can be viewed as a generalization of maximum likelihood but is anathema to many because of its assignment of probability distributions to parameters that are not the products of random processes. It targets probability distributions that represent the current understanding of the realities and uncertainties involved. Nonparametric approaches such as bootstrap and lasso have traditionally been shoehorned into the frameworks of hypothesis testing and interval estimation, but in recent years, the machine learning approach has focused not on those classical problems but rather on pure prediction. They aim to reduce the assumptions (used to represent the current understanding of the realities and uncertainties) involved and to enhance the identification of procedures with seemingly good properties. The decision on what information to combine is often dictated by probability models or inferential algorithms that are largely chosen by convention. This occurs for basic users who are taught to use *t*-tests for continuous data (group variances assumed to be common, to give a combined variance estimate with more degrees of freedom), $\chi^{2}$ tests for discrete data (various choices about common parameters when defining expectations to test consistency with), linear regression models (assuming all observations have common slopes given that the explanatory variables fit as well as a common standard deviation), Cox models for survival data (common proportional hazard function assumed so that it cancels out), etc., but even experienced statisticians often seem unclear about the choices made regarding which information to combine in their data analysis.

Even amid the diversity of statistical methods and philosophies, all these approaches involve the amalgamation of evidence. This is true from the simplest models of random sampling and independent identically distributed data; to slightly more complex models with hierarchical, time-series, and spatial structures; to multistage deep learning algorithms that combine thousands of predictors or features. Even something as basic as Fisherian *p*-values or likelihood-ratio testing can be seen as ways to use the accumulation of data—that is, the piling-up of evidence—to draw increasingly certain conclusions; the integration of likelihood, although considered Bayesian, can also be interpreted more generally [8].

Modern data science has moved away from a mathematical framework of hypothesis testing and model building, toward a computationally focused environment of prediction, external validation, and reproducibility [9]. From this perspective, we can think of evidence being combined not to evaluate theories but to form more effective predictions; however, probability modeling can still be useful in constructing procedures that combine information efficiently while pointing to potential areas of sensitivity where predictions of interest are strongly influenced by particular decisions regarding pooling, partial pooling, or exclusion of evidence.

It has been said that the most important aspect of a statistical method is not what it does with the data but rather what data it uses. From this perspective, the power of Bayesian, regularization, and machine learning methods lies in their ability to incorporate large amounts of data into analysis and decision-making.

At the same time, as datasets become larger and more diverse, there is an increasing need to model and adjust for differences between samples (that is, available data) and populations, and between treatments and control groups in causal analysis. Amalgamation of evidence is important but it is not trivial; it is not just a matter of throwing data into a blender. One must evaluate data quality to decide what to include. Or, more generally, one must weigh and adjust data in light of what is known about the quality and representativeness of measurements and in light of the consistency of different data sources with available research hypotheses. Implicitly, these procedures can be seen as derived from different probabilistic data-generating models and prior distributions, but in our discussion, we focus on the information included in data analysis, not the algorithms used to construct inferences or the models underlying these algorithms.

Some of the fiercest debates in statistical theory and practice involve the use of prior information. For example, the well-respected statistician David Cox wrote the following:

“There are situations where it is very clear that whatever a scientist or statistician might do privately in looking at data when they present their information to the public or government department or whatever, they should absolutely not use prior information because the prior opinions on some of these prickly issues of public policy can often be highly contentious with different people with strong and very conflicting views.”

[10]

We expressed disagreement, pointing to a problem on “the politically controversial problem of reconstructing historical climate from tree rings”:

“We have a lot of prior information on the processes under which tree rings grow and how they are measured. I don’t think anyone would want to just take raw numbers from core samples as a climate estimate! All the tools from Statistical Methods for Research Workers won’t take you from tree rings to temperature estimates. You need some scientific knowledge and prior information on where these measurements came from.”

[11]

Cox had decades of applied experience and would surely have agreed that prior information, in the form of physical/biological models, is essential to making climate-related decisions based on tree rings, and we are sure he would also have agreed that such models involve inevitable subjective choices. Rather, we believe Cox was concerned about the way that Bayesian methods can be abused, what one might call the “moral hazard” involved in a statistical method, in which all modeling decisions are up for grabs. In addition, there is concern that, in most settings, including the tree-ring example, expressing prior information as probability distributions can lead, paradoxically, to a false sense of certainty. Hence, the preference of Cox and others for the inclusion of prior information in a more piecemeal, case-by-case manner. From this perspective, the smoothness and apparent all-encompassing nature of Bayesian inference is itself a hazard.

The paradox is that flexibility is required to combine evidence from diverse sources, but if that flexibility is abused, the ultimate conclusions of the analysis can be dictated by the analyst rather than by the data. Perhaps default methods for combining evidence from diverse sources will be too hazardous. This is a concern with Bayesian inference with overconfident priors and with classical inference when “p-hacking”, “researcher degrees of freedom”, and “the garden of forking paths” allow users to find statistical significance from virtually any dataset [12]. And there is also the choice of what statistical method to use, a decision that is typically not based on statistical evidence [13]. We offer no general solution here but we think it useful to formulate all statistical methods as data aggregators of one sort or another and to be open about the evidence used to form any particular statistical conclusion—and also the available evidence that, for one reason or another, has been “left on the table” and is not yet incorporated into our inferences.

Beyond this, when we move beyond simple textbook examples of experimentation and sampling, there is typically no default analysis available. There is no general way to decide how to choose, combine, and transform regression predictors or features in a predictive model, and many scientific problems inherently involve the integration of information from different sources, for example, medical research interpreting clinical trial data in the light of biological models; or climate modeling combining data from tree rings, historical temperature data, and physical modeling; or election forecasting combining information from state polls, national polls, and forecasts based on economic and political conditions.

## 3. Amalgamation of Evidence in the Scientific Process

Statistical modeling typically focuses on a particular set or stream of data, which leads to some inference or decision. But it can also be helpful to think more “sociologically” of an evolution-like mechanism involving thousands of research hypotheses, millions of scientists, and processes of publication, publicity, career rewards, and replication, which lead not just to specific conclusions but also to strands of research, subfields, and allocations of research effort, as C. S. Peirce might have put it [14], communal science that is (and remains) profitable. In particular, in the field of psychology, there has been much recent discussion on the replicability (or lack thereof) of published research claims, and similar concerns have been raised in medical research. As Peirce [15] wrote,

“The theory here given rests on the supposition that the object of the investigation is the ascertainment of truth. When the investigation is made to attain personal distinction, the economics of the problem are entirely different. But that seems to be well enough understood by those engaged in that sort of investigation.”

But the current de facto procedure, in which studies are summarized by statistically significant estimates, has technical problems of bias and inefficiency even if we assume all researchers are acting altruistically.

Considering the entire academic research enterprise—the processes of peer reviews, publications, replications, and meta-analyses—as a grand collective effort of information aggregation, we join a long string of concerns from Peirce through [16] in seeing major problems with incentives and structure, and where simple technical fixes such as weighting studies by appraised quality can be disastrous [17]. Smaldino and McElreath [18] offer a simplified but suggestive model of problems with the current system of incentives and publications. On the one hand, the diversity of research labs must represent a strength, a potential escape from the groupthink that is associated with central planning. But, from the statistical standpoint, much information is lost by dividing our data into small pieces and summarizing each by a *p*-value. This would be an inefficient procedure even if *p*-values were computed as described in the textbooks based on pre-specified tests, but problems of drastic overestimation of effect sizes (type M or “magnitude” errors) become even worse given the documented ability of researchers at all levels to attain statistical significance virtually at will. Systematic overestimation of effect sizes creates a vicious cycle in which new studies are incorrectly anticipated as having a high probability of being successful [19], leading to further data whose significance is overstated.

A cleaner approach would be to analyze larger datasets directly, not by post-processing published estimates and *p*-values but by modeling larger and more diverse sets of raw data. This gives direct access to more efficient statistical analyses and also more ability to check model assumptions. Fisher unfortunately may have undermined the appreciation of this with his claim:

“It is usually convenient to tabulate its [the likelihood’s] logarithm, since for independent bodies of data such as might be obtained by different investigators, the “combination of observations” requires only that the log-likelihoods be added.”

[20]

This is technically correct if the data-generating model (which defines the likelihood) is never questioned or assessed—but it should be. To do this adequately, one needs all the individual raw data from all studies. Here, we quickly add that the prior’s logarithm need only be added to the likelihood’s logarithm to start a Bayesian analysis. Like the data-generating model, the prior also needs to be questioned or assessed. Again, we see a statistical and societal advantage to explicit recognition that inference arises from amalgamation of evidence, and more openness to the sources of this evidence and possible biases.

To step back from data analysis to the scientific enterprise more generally, various specific reforms of science have been proposed, including post-publication reviews, preregistered replications, and publication/career credits for data quality (rather than just for novelty and statistical significance). We find it helpful to follow Peirce and think of these as steps in a larger process, rather than just merely attempts to minimize false positives in isolated studies. This quote from Peirce might suffice: “I [Peirce] do not call the solitary studies of a single man a science. It is only when a group of men, more or less in intercommunication, are aiding and stimulating one another by their understanding of a particular group of studies as outsiders cannot understand them, that I call their life a science.” We also include two longer passages:

“Science is to mean for us a mode of life whose single animating purpose is to find out the real truth, which pursues this purpose by a well-considered method, founded on thorough acquaintance with such scientific results already ascertained by others as may be available, and which seeks cooperation in the hope that the truth may be found, if not by any of the actual inquirers, yet ultimately by those who come after them and who shall make use of their results.”

(also in [21])

“But what I mean by a “science” (*…*) is the life devoted to the pursuit of truth according to the best-known methods on the part of a group of men who understand one another’s ideas and works as no outsider can. It is not what they have already found out which makes their business a science; it is that they are pursuing a branch of truth according, I will not say, to the best methods, but according to the best methods that are known at the time. I do not call the solitary studies of a single man a science. It is only when a group of men, more or less in intercommunication, are aiding and stimulating one another by their understanding of a particular group of studies as outsiders cannot understand them, that I call their life a science. It is not necessary that they should all be at work upon the same problem, or that all should be fully acquainted with all that it is needful for another of them to know; but their studies must be so closely allied that any one of them could take up the problem of any other after some months of special preparation and that each should understand pretty minutely what it is that each one of the other’s work consists in; so that any two of them meeting together shall be thoroughly conversant with each other’s ideas and the language he talks and should feel each other to be brethren.”

## 4. Connections to the Philosophy of Science and the History of Statistics as a Quest for Principled Amalgamation

Statistical science has evolved from the growing awareness, extraction, and assessment of commonness amid diversity. Not only can physical laws (or, as social scientists say, “law-like relationships”) be uncovered from noisy data, in the manner of Gauss, Laplace, and their followers, such as Airy [22], variations can be categorized and thought of as forms of commonality. This was a key insight of Galton, Pearson, and other statisticians who in the late 19th century applied the concept of probability distribution to biological variation. We have argued that, in recent years, this insight has been oversold, now that researchers have the demonstrated ability to extract large, statistically significant, spurious, and unreplicable findings from just about any dataset; that said, from a historical point of view, the idea that variation can itself be quantified is central to any statistical understanding of modern social and biological sciences.

Here, we focus on methods of quantifying commonness among different empirical studies and their reported observations. Commonness refers to studies aimed at the same target (aspect of reality) as well as qualitatively similar evidence of that target, hopefully varying only in precision, which can be readily assessed. On the other hand, qualitatively different data sources can vary in their biases, which may be very difficult to assess and properly correct so that something is actually common. Terms from psychometrics that make the same distinctions as bias and precision do here would be validity and reliability.

Awareness of commonness can lead to an increase in evidence regarding the target; disregarding commonness wastes evidence; and mistaken acceptance of commonness destroys otherwise available evidence. It is the tension between these last two processes that drives many of the theoretical and practical controversies within statistics. A concrete but simple example that demonstrates practical controversies nicely would be the situation depicted in the Wiki entry on Simpson’s paradox [23]. The illustration of the quantitative version: a positive trend appears for two separate groups (blue and red), whereas a negative trend (black, dashed) appears when the groups are combined.

The illustration clearly depicts an underlying reality of exactly the same positive trend for two groups (both slopes equal to 1) that happen to have different intercepts, one at about 5 and the other at −7. A default application of regression modeling using the eight data points displayed in the illustration would likely specify a single intercept, slope, and standard deviation parameter. The incorrect single intercept here is a mistaken acceptance of commonness, which destroys the evidence for common positive slopes by providing a single negative slope estimate of roughly −0.6, in addition to providing a single incorrect intercept estimate of about 9. Specifying the correct commonness here—that of separate intercepts but a single common slope and single standard deviation parameter—captures (all the evidence for) the correct intercept and slope with no actual error, with the correct estimates of the intercepts of 5 and −7, the slope of 1, and the single standard deviation of 0. With realistic data, there are observation errors and the specification of separate intercepts, but incorrectly separate slopes and a common standard deviation parameter can waste evidence, providing two different slope estimates randomly varying around 1, and a biased-downward estimate of the standard deviation. One might further ask or question why the assumption of a common standard deviation was being made. Simply convenience?

This simple contrived example from Wiki illustrates a lack of concern regarding the need to accurately represent reality (correctly specifying common and non-common parameters) as accurately as possible, or at least well enough for statistical procedures to provide reasonable answers. The usual training in statistics likely suggests the default use of a common intercept in multivariate regression analysis as well as the occasional need to consider interactions (with the statistical custom of always specifying separate intercepts for interaction terms—the lower-order terms). But here, without interaction, evidence is destroyed, while with interaction, it is wasted. The result is misleading descriptive or predictive inference in the former scenario and inefficient descriptive or predictive inference in the latter.

Better descriptive or predictive inferences come from better underlying representations of reality. For descriptive or predictive inference, reality, as it is now (and likely to persist), is the only reality that needs to be represented well enough. On the other hand, for causal or transportable inference, both current realities and how those can be changed (causal) or local and remote realities (to transport between) need to be well represented [24]. For instance, randomization creates two similar realities, one of which can presumably be modified in a hopefully simple manner (though maybe not as simple as an additive effect). Causal and transportable inference is, of course, much more challenging, but this complexity should not lead to disregarding the importance of representing a single current reality accurately for descriptive or predictive inference.

Statistical science historically emerged out of the conjecture, assessment, and reasoned acceptance of the commonness of observations made by different members of the community of astronomers. Among a set of apparently related observations, some combination was conjectured to be better than just enumerating the set, but a justification for how to weigh observations, whether repeatedly made by the same astronomer or by different astronomers, was completely lacking and desperately sought. Astronomers and others would often reflect on how to determine which dataset was the best (thus implicitly assigning weights of 0 to all the remaining data); anticipating that was the obvious solution, but they had yet to learn that, as Stigler [25] put it, “the details of individual observations had to be, in effect, erased to reveal a better indication than any single observation could on its own”. In the modern world of social media, we similarly speak of the wisdom of crowds, an idea which is often illustrated using an example by Galton [26].

The problem of information aggregation has attracted the attention of the brightest minds of the time, including mathematicians and philosophers such as Laplace and Gauss. Its resolution came from recognizing a common object being measured by all and the reasonableness of a common error probability model for all—regardless of whether the observations were made by the same or different astronomers. This involved a model for both the common target of reality (the aspect of reality the observation was attempting to capture) and a common observational error that is the same for all. According to [27], it was the idea of “dealing with observations made by various other observers under different conditions—that actually ‘spurred’ on the development”. The probabilistic error model, along with the willingness to use it on data from multiple sources, was the key technological insight needed. In gambling, probability models provided a means to determine the best bet regarding outcomes from games and devices that had common chance outcome mechanisms; in contrast, in astronomy, the error probability model representing common errors provided a means to determine the best combination for a target taken as common and, hence, the best weights for the combination of observations [28,29]. In much of statistical practice, probability models provide a formal mathematical basis for amalgamating and assessing commonness, which then sets out the best combinations for various purposes. For overviews and historical accounts; see [25,30,31]. More recently, machine learning methods have shifted to more algorithmic, less model-based approaches—not from any perceived defect with the probability models but rather for computational reasons when dealing with “big data”—but, again, the principle remains that data from different sources can and will be pooled in a single procedure (unless trivially based on single observations).

A repeated broadening of what was considered common can be briefly outlined here, starting with the initial recognition of a common object being measured and the reasonableness of a common error model that implied the weights for the best combination. The next step involves extending or revising this to still include a common object being measured but now with a differing error model, i.e., one that allows for a source of error that affects all observations taken on that day, yet represented as being drawn from a common distribution of error distributions (a recognition of commonness at a higher level). This extension/revision implies different weights for the best combination. Earlier, in a different context than astronomy (ratios of male to female births in different cities), the reasonableness of a common error model was kept but the object being measured itself was not taken as common; instead, it was conjectured/represented as being a draw from a common distribution of objects. That is, the objects being measured were allowed to vary but were aligned with being drawn from a shared probability distribution. At this point, the purposeful design (or induction of commonness) in the observations’ underlying distributions emerges. An early instance was Peirce’s recognition that random sampling and random assignment of treatments induce a common distribution for the sample and population, or treatment and control group. Nowadays, we might frame all these issues using multilevel models with variance at the observation level and, in the astronomy context, variance components for individual measurement methods, astronomers, and other factors that could induce systematic error.

In Bayesian inference, the prior density is just multiplied by the factors of the likelihood, quantifying the information coming from the data (conditional on the assumed class of models). The prior can then be seen mathematically as just one more data point. To make this absolutely clear, each observation defines a likelihood (the probabilities of observing that very observation for the various parameter values the parameters can take), and the study likelihood is a product of those single observation likelihoods (conditioned on other observations if observations are not independent). The posterior is proportional to the prior multiplied by the combined study likelihood. This multiplication can be rearranged and re-expressed in any way that does not change it. Taking logarithms, the log posterior is proportional to the sum of the log prior and all the individual log-likelihoods—a “weighted combination” with the “weights” determined by the functional form of the prior density and individual likelihoods. Some authors object to the prior being in this combination, using what could be seen as an apples and oranges argument, arguing that now what is being amalgamated is of a different nature. Reid and Cox [32] expressed concerns with “merg[ing] seamlessly what may be highly personal assessments with evidence from data possibly collected with great care”, instead using prior information “largely or entirely qualitatively”. We disagree and see this seemingly outright refusal to consider possible representations of commonness between prior and observations as simply “blocking inquiry” by disallowing a potentially profitable scientific representation of the unknown that may well be a “powerful aid to the formation of true and fruitful conceptions”, to paraphrase Peirce. For the present paper, it is not necessary to resolve this disagreement but just to point out that it can viewed as a question of amalgamation of evidence rather than as a dispute of objectivity vs. subjectivity, which is how Bayesian/non-Bayesian debates are often framed; for further discussion on this topic, see Gelman and Hennig [33].

From our perspective, one can “interpret the parameter prior in a frequentist way, as formalizing a more or less idealized data generating process generating parameter values” [33]. One of the earliest to concretely express this view was Francis Galton, who constructed a physical machine to clearly demonstrate both parameter and observation generation. It involved a two-stage quincunx. The top level represents the generation or setting of the unknown parameter (the prior) and the second level is the generation of a single noisy observation of each observed object’s value (the data-generating model or likelihood). By tracing back from a chosen value of noisy observations (the slot the pellet ended up in) and identifying all the various values of unknown parameters that had generated them, a crude sample from the posterior is identified and obtained. Though clunky and limited (just a single unknown parameter with a single observation from each), it fully demonstrates how Bayesian inference uses probability-generating models, both for parameter values and observations, to amalgamate commonness between observations and then those observations and the prior).

There are real risks of taking things as common, in a sense that—in reality—they are not, whether between the parameter-generating process and the data-generating process or among the data-generating process for different observations themselves. We use the phrase “conjecture, assessment, and reasoned acceptance of the commonness” to emphasize that. But similar scientific judgment is required in deciding how to combine measurements—the “likelihood” part of the model—and we do not see the risks of model errors as being qualitatively different when considering data-combination rules, such as when considering how to express prior information; see Evans [34] on this point.

Bayesian models “domesticate” uncertainty by turning it into (probabilistically represented) variation; in economic jargon, they convert Knightian uncertainty into quantifiable risk. Such procedures gain statistical efficiency at the cost of making mathematical assumptions about distributions and, more importantly, the independence of error terms (strong replication), thus inducing skepticism among many potential users; however, alternative approaches that seem to avoid such assumptions typically perform information aggregation in some other way, for example, by avoiding pooling across data sources but then averaging over time. In nearly any situation where a decision needs to be made, *some* choices need to be made regarding the pooling of data.

Comparing the technique of nearest neighbors to linear regression will help clarify what we mean by unavoidable choices being made for pooling. For simplicity, consider a single *x* variable and its role in predicting a single *y* variable. A linear regression model conjectures a single common intercept and common slope for predicting the expected value of *y* from all values of *x* as well as a common standard deviation parameter. The probability model for all observations is taken to be normal(a+bx,σ) and all observations provide evidence for just three parameters. In contrast, nearest neighbor regression tries to avoid specifying any commonality of expected values of *y* for differing values of *x*, instead allowing expected values to vary arbitrarily by neighborhood. The technique identifies these neighborhoods from the observations and takes averages only within neighborhoods (never across). It usually specifies the sizes of these neighborhoods. Taking the size of the neighborhoods as 2 requires that a single nearest neighbor is found for every observation and assumed to have the same expectation—referred to as ${NN}_{1}$. Nearest neighbors do not actually avoid specifying some commonness, however, as ${NN}_{0}$ is not taken as an acceptable procedure (having no neighbors, all observations must be taken as islands on their own). So, commonness of expectation between at least two observations is enforced. Then, to achieve better “good” properties, commonness is allowed over a larger number of observations depending on the dataset—referred to as ${NN}_{k}$. Additionally, a common variance is usually assumed between neighborhoods (a secondary feature). To derive combinations based on more than one observation, and to obtain variance estimates from more than a few isolated points in each neighborhood, you treat non-common points and non-common parameters “grudgingly” as common—simply to improve the properties for estimating what you can.

This alternative approach to statistics avoids relying on probability models, instead aiming for procedures that work well under weak assumptions; for example, instead of assuming a distribution is Gaussian, you would just want the procedure to work well under some conditions on the smoothness of the second derivative of the log density function. These approaches have also evolved in astronomy, with Legendre developing least squares regression without requiring the probability-generating models that Gauss had assumed and used to obtain the exact same technique.

Instead of requiring probability model assumptions, this approach requires a choice of good properties (why minimize squared error?) over a class of problems to be dealt with (where values of unknowns are constrained in some way, such as being linear in regression or proportional hazards in survival analysis). Probability models make representations that aim to capture certain aspects of reality that cannot be directly assessed but do allow for indirect evaluations of their adequacy. On the other hand, alternative approaches choose properties to be optimal for a given class of applications (e.g., applications having linear expectations or proportional hazards) with no direct justification for the goodness of the property nor guarantees of a particular application belonging to that class; that is, with no way to assess the goodness of the property or belonging within that appropriate class, without making some representation of reality to average or maximize over.

Given sufficient flexibility, data aggregation can always be seen as appropriate, but if the data to be combined are too different—and if there is no good model to bridge these differences—there will be little or no practical gain from pooling, and indeed, there can be a risk if analysts use inappropriately strong models that do not sufficiently account for variation among data sources.

With regard to exactly when observations have something in common amongst them so that aggregation can be applied to useful effect, there is always some judgment involving “replication (or exchangeability) on some level by the statistician” (Gelman and Hennig, 2017). For a replication to be a true replication and not a mere duplication, there must not be complete dependence, and for a replication to be strong, there must be as much independence as possible. Often data-analytic procedures are set up in terms of observations that can be taken as independent under reasonable assumptions. It is through these unit-of-analysis contributions that we wish to understand how to conjecture, extract, and assess commonness. In astronomy, the units of analysis were simply individual observations and they were understood as being independent.

An extreme case often arising in social science is when differing scales (for example, aggressiveness, anger, etc.) are used for assessing treatment effects in different randomized experiments. It can be challenging, especially given what is reported in such studies, to specify probability-generating models for these different outcomes that have common parameters. This points to the interplay between the design of experiments, data collection, and analysis, as expressed for example by Cox [35]. Cleaner data collection puts less of an analysis burden; conversely, the sorts of “big data” that arise from social media, etc., are messy and require more assumptions in order to make causal inferences and generalize from sample to population. This in turn increases computational requirements, both from the sample size and model complexity, and helps explain why much of the work of modern applied and theoretical statistics centers on algorithms and computing. Again, this is all happening within the context of information aggregation; see, for example, Li, Srivastava, and [36].

By aiming to accurately represent reality, and recognizing that an aspect of this reality being common is part of what validates commonness, we align ourselves with the broader philosophical community as defined by Peirce, Ramsay, and others. As we stated elsewhere [33], “Although there is no objective access to observer-independent reality, we acknowledge that there is an almost universal human experience of a reality perceived as located outside the observer and as not controllable by the observer. We see this reality as a target of science, which makes observed reality a main guiding light for science. We are therefore ‘active scientific realists’ in the sense of [37], who writes: ‘I take reality as whatever is not subject to one’s will, and knowledge as an ability to act without being frustrated by resistance from reality’ and ‘Active scientific realism implies that finding out the truth about objective reality is not the ultimate aim of science, but that science rather aims at supporting human actions’”. We add here that we strive for more than just not being frustrated by resistance from reality; rather, we want our findings and claims that aim at truth to be “beliefs which succeed for reasons connected to the way things are” [38].

The classical view of statistics, briefly mentioned before, primarily about procedures to obtain estimates, tests, confidence intervals, etc. with certain good properties (often common properties for all possible unknowns), has limitations when moving beyond simple settings. We believe scientific research would be more effective if statistics was viewed instead as primarily about conjecturing, assessing, and adopting idealized representations of reality, predominantly using probability-generating models for both parameters and data that can make the most out of commonness, for example, using hierarchical models with group-level predictors so that unexplained group-level variance is low and more information can be pooled from different sources. It seems to be already widely supported for probability-generating models for data “[providing an] explicit description in the idealized form of the physical, biological, ...data generating process”, which is essentially “to hypothesize a data generating mechanism that produces observations as if from some physical probabilistic mechanisms” [32]. We have argued that limiting probability-generating models just for data while banning them for parameters is too restrictive for much of science.

Our belief in the efficacy of information aggregation, using continuous parameters to determine the level of partial pooling, is supported by a belief that reality—though never directly accessible—is continuous, in which different experiments, treatments, and outcomes are connected somehow rather than distinct severed islands on their own. Differing considerations and purposes can then be brought to bear on what best combinations (estimates, summaries) follow. From a slightly different direction, Tibshirani [39] argues that enforcing sparsity is not primarily motivated by beliefs about the world, but rather by benefits such as computability and interpretability, indicating how considerations other than correspondence to reality often play an important role in statistics and, more generally, in science. Tibshirani’s view fits squarely within the alternative “classical” or non-Bayesian approach, in which techniques are chosen based on various robust operational properties rather than being viewed as approximations of reality. With this in mind, when we indicate that we consider generating models as an idealization, we need to point out that they could be, in fact, just ‘fictions’—useful ‘fictions’ if they lead to an ability to act without being frustrated by resistance from reality. Sometimes, fiction does turn out to be connected with how things truly are. But, if their connections are accidental, with anything more than just in the short term, we suspect that they will not be as profitable for scientific practice, as, by definition, science (continuously) tries to represent reality accurately, or at least less wrong.

## 5. Conclusions

The foundations of statistics remain controversial, even among its leading practitioners, unlike fields such as biology, chemistry, or physics, which no longer experience such foundational disputes. In many ways, statistics looks more like social sciences, such as sociology, economics, and political science, which are riven by deep ideological divisions, but with the difference that statistics is a field of mathematics and computing in which ideology does not seem to play any obvious role. However, mathematics and computing just define and implement the tools (where there is much agreement), the purposes for which these tools should be used, and the interpretations of the results they produce in specific applications, extending beyond just mathematics and computing (and here, there is little agreement).

Whatever the historical sources and ultimate resolutions of the debates within the field of statistics, we see the combination of evidence as central to *any* statistical method, and we view methods as stronger to the extent that they can incorporate diverse sources of information, weighting, or adjusting appropriately to account for inevitable problems of data quality and representativeness.

Furthermore, we view statistical concepts of data integration, along with the quantification of uncertainty and variation, as central to understanding and reforming the currently broken system of scientific publication and promotion.

Finally, all these concerns relate to a longstanding skeptical tradition in the philosophy of science. Ironically, various modern abuses of statistics, such as the pursuit of statistical significance or, more generally, the deterministic thinking that leads researchers to establish certitude beyond the capabilities of their data, arise from skeptical ideas in statistics, such as Fisher’s warnings about over-interpreting chance variation or the Neyman–Pearson–Wald rigorizing of certain stylized statistical decision problems.

When amalgamating evidence, we typically are at least one step beyond the available theory—it only *feels* like amalgamation if it cannot be done automatically—but we should not let this stop us from trying. It is through recognizing, formalizing, and modeling our attempts at combining information—and by recording and learning from our failures—that we will do better.
